# Removal of the E^rns^ RNase Activity and of the 3′ Untranslated Region Polyuridine Insertion in a Low-Virulence Classical Swine Fever Virus Triggers a Cytokine Storm and Lethal Disease

**DOI:** 10.1128/jvi.00438-22

**Published:** 2022-06-27

**Authors:** Miaomiao Wang, José Alejandro Bohórquez, Sara Muñoz-González, Markus Gerber, Mònica Alberch, Marta Pérez-Simó, Xavier Abad, Matthias Liniger, Nicolas Ruggli, Llilianne Ganges

**Affiliations:** a World Organisation of Animal Health (WOAH) Reference Laboratory for Classical Swine Fever, Instituto de Investigación y Tecnología Agroalimentaria, Centre de Recerca en Sanitat Animal, Barcelona, Spain; b Unitat mixta d’Investigació Instituto de Investigación y Tecnología Agroalimentaria-Universitat Autònoma de Barcelona en Sanitat Animal, Centre de Recerca en Sanitat Animal, Campus de la Universitat Autònoma de Barcelona, Barcelona, Spain; c Instituto de Investigación y Tecnología Agroalimentaria, Programa de Sanitat Animal, Centre de Recerca en Sanitat Animal, Campus de la Universitat Autònoma de Barcelona, Barcelona, Spain; d The Institute of Virology and Immunology, Mittelhäusern, Switzerland; e Department of Infectious Diseases and Pathobiology, University of Bern, Bern, Switzerland; Loyola University Chicago

**Keywords:** classical swine fever virus, virulence factors, innate and adaptive immunity, disease severity, pigs, vaccine designs, cytokine-mediated symptoms, diagnostic tools and vaccine strategies, tonsil, viral replication, viral spread

## Abstract

In this study, we assessed the potential synergistic effect of the E^rns^ RNase activity and the poly-U insertion in the 3′ untranslated region (UTR) of the low-virulence classical swine fever virus (CSFV) isolate Pinar de Rio (PdR) in innate and adaptive immunity regulation and its relationship with classical swine fever (CSF) pathogenesis in pigs. We knocked out the E^rns^ RNase activity of PdR and replaced the long polyuridine sequence of the 3′ UTR with 5 uridines found typically at this position, resulting in a double mutant, vPdR-H_30_K-5U. This mutant induced severe CSF in 5-day-old piglets and 3-week-old pigs, with higher lethality in the newborn (89.5%) than in the older (33.3%) pigs. However, the viremia and viral excretion were surprisingly low, while the virus load was high in the tonsils. Only alpha interferon (IFN-α) and interleukin 12 (IL-12) were highly and consistently elevated in the two groups. Additionally, high IL-8 levels were found in the newborn but not in the older pigs. This points toward a role of these cytokines in the CSF outcome, with age-related differences. The disproportional activation of innate immunity might limit systemic viral spread from the tonsils and increase virus clearance, inducing strong cytokine-mediated symptoms. Infection with vPdR-H_30_K-5U resulted in poor neutralizing antibody responses compared with results obtained previously with the parent and RNase knockout PdR. This study shows for the first time the synergistic effect of the 3′ UTR and the E^rns^ RNase function in regulating innate immunity against CSFV, favoring virus replication in target tissue and thus contributing to disease severity.

**IMPORTANCE** CSF is one of the most relevant viral epizootic diseases of swine, with high economic and sanitary impact. Systematic stamping out of infected herds with and without vaccination has permitted regional virus eradication. However, the causative agent, CSFV, persists in certain areas of the world, leading to disease reemergence. Nowadays, low- and moderate-virulence strains that could induce unapparent CSF forms are prevalent, posing a challenge for disease eradication. Here, we show for the first time the synergistic role of lacking the E^rns^ RNase activity and the 3′ UTR polyuridine insertion from a low-virulence CSFV isolate in innate immunity disproportional activation. This might limit systemic viral spread to the tonsils and increase virus clearance, inducing strong cytokine-mediated symptoms, thus contributing to disease severity. These results highlight the role played by the E^rns^ RNase activity and the 3′ UTR in CSFV pathogenesis, providing new perspectives for novel diagnostic tools and vaccine strategies.

## INTRODUCTION

Classical swine fever (CSF) is one of the most highly contagious viral diseases that affect swine worldwide (1). The disease is caused by the CSF virus (CSFV), a member of the *Pestivirus* genus within the *Flaviviridae* family. CSF has been eradicated in central Europe but remains endemic in some regions of South and Central America and Asia and in the Caribbean (2). The most recent outbreaks have been reported in Colombia, Japan, Brazil, Bhutan, Ecuador, Russia, and Thailand. Notably, the recent outbreak of CSFV in Japan after 26 years of CSF-free status shows that CSFV remains a reemerging threat to pork production worldwide (2, 3). Moreover, long-term prevalence of CSFV in regions of endemicity has led to a broad range of clinical outcomes, from acute to chronic and subclinical, including persistent infection (1, 4).

CSFV is an enveloped virus with a single-stranded, positive-sense RNA genome of approximately 12.3 kb. The RNA carries a single open reading frame (ORF) that encodes a polyprotein flanked by 5′ and 3′ untranslated regions (UTRs). The translated polyprotein is processed into four structural proteins (C, E^rns^, E1, and E2) and eight nonstructural proteins (N^pro^, p7, NS2, NS3, NS4A, NS4B, NS5A, and NS5B) (5, 6).

The pestivirus proteins N^pro^ and E^rns^ interfere with the host’s innate immune responses by counteracting type I interferon (IFN) synthesis, with implications in pathogenesis (7–10). Functional E^rns^ possesses a unique intrinsic RNase activity (11) which acts as an antagonist for IFN induction by degrading viral single- and double-stranded RNA conditionally in the extracellular space and in endocytic compartments (12, 13).

Previous studies showed that the low-virulence CSFV field isolate Pinar de Rio (PdR), which can cause chronic and persistent forms of CSF, contains a unique and uninterrupted 36-uridine (poly-U) insertion in the 3′ UTR (4, 14, 15). This poly-U insertion has been associated with activation of immunity and reduction of virulence in piglets (16). Likewise, a previous study also revealed loss of pathogenicity and replication capacity in pigs when the E^rns^ RNase function was abrogated in the low-virulence CSFV PdR strain carrying the poly-U (17). Therefore, in the present study, we investigated the potential synergistic effect of the E^rns^ RNase activity and the poly-U insertion in the 3′ UTR on the regulation of innate and adaptive immunity and its relationship with CSF pathogenesis in pigs. To this end, a reverse genetics approach was used to analyze the phenotype of a double mutant of the low-virulence PdR strain in which the RNase activity of E^rns^ was knocked out and the aforementioned 36 uridines of the 3′ UTR were replaced with 5 uridines common to most CSFV isolates at this position. We analyzed the replication of this virus in monocyte-derived macrophages (MDM) and the IFN-α induction in plasmacytoid dendritic cells (pDC) and determined pathogenicity, replication, cytokine induction, and immune responses in 5-day- versus 3-week-old pigs.

## RESULTS

### Inactivation of the E^rns^ RNase and deletion of the poly-U sequence in PdR did not impair virus replication in cell culture compared with that of the parent virus.

**FIG 1:**
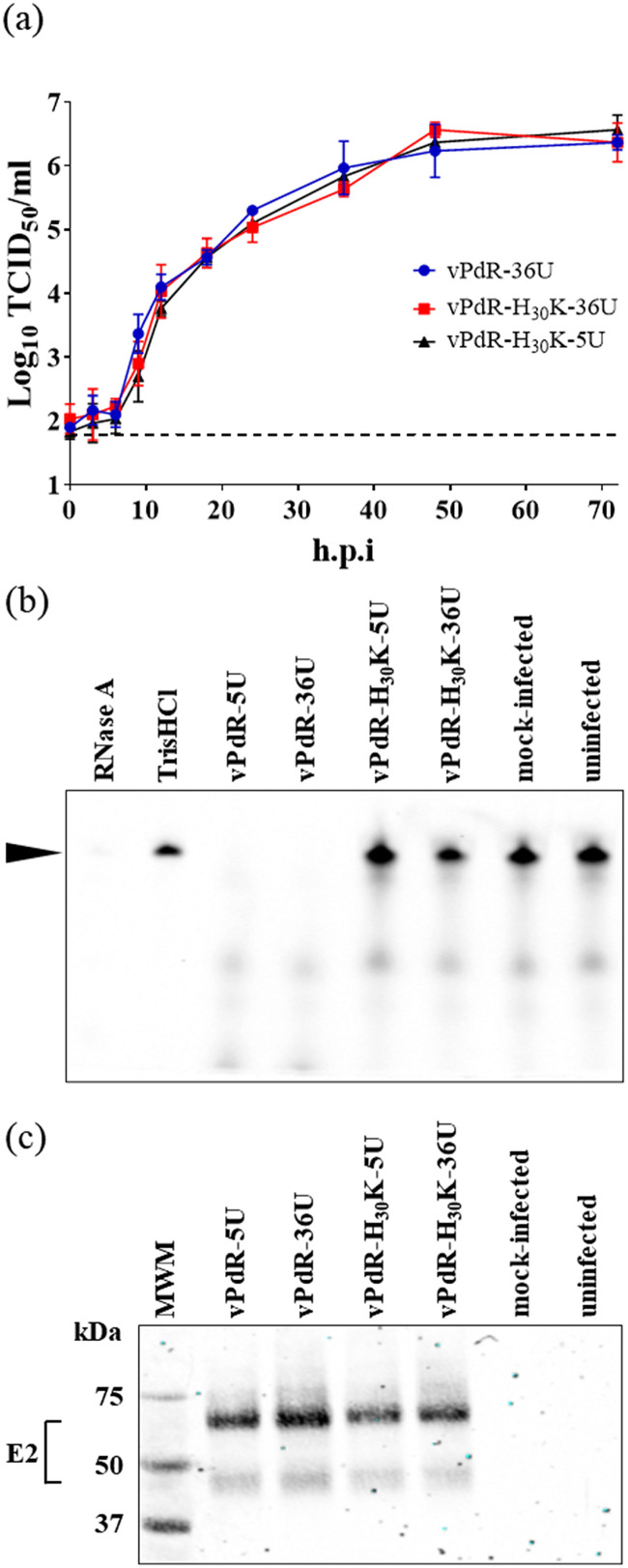
Kinetics of replication and RNase activity of the different mutant vPdR-derived viruses. (a) Porcine MDM were infected in parallel multiwell plates (one per time point) with either vPdR-36U, vPdR-H_30_K-36U, or vPdR-H_30_K-5U and incubated for the indicated times. The mean virus titer from triplicate infections was determined in SK-6 cells; error bars represent the standard deviations. (b) In order to determine the E^rns^ RNase activities of the different mutant viruses, SK-6 cells were infected, mock treated, or left untreated for 40 h. After total cell protein harvest by hypotonic lysis, the Dy-781-O1-RNA probe (black arrowhead) was incubated with EMEM containing RNase A or with the cell extracts and, finally, separated by urea PAGE. (c) Protein extracts from parallel infected cell cultures were separated by SDS-PAGE under nonreducing conditions and analyzed by Western blotting and immunodetection with the anti-E2 (HC/TC26) and anti-β-actin MAbs, using the goat anti-mouse IRDye680 as secondary antibody.

In order to study the synergistic role of the poly-U sequence found in the 3′ UTR and the E^rns^ RNase activity of CSFV PdR, a double mutant of the PdR strain (vPdR-H_30_K-5U) that lacks both determinants was constructed as described in Materials and Methods. The high specific infectivity of the RNA transcripts at rescue and the nucleotide sequence analysis of the vPdR-H_30_K-5U virus passaged twice in PEDSV.15 cells confirmed the functionality and identity of the double mutant. The replication kinetics of the vPdR-H_30_K-5U virus did not differ from the replication of the parent vPdR-36U and the RNase knockout vPdR-H_30_K-36U mutant in freshly prepared porcine monocyte-derived macrophages (Fig. 1a). The absence of any detectable E^rns^ RNase activity of the vPdR-H_30_K-5U virus and of the vPdR-H_30_K-36U virus described previously was verified in SK-6 cells and compared with those in the parent vPdR-36U and vPdR-5U control viruses (Fig. 1b), while viral protein expression was comparable for the 4 viruses (Fig. 1c).

### The vPdR-H_30_K-5U double mutant strongly activated the IFN-α response in pDC.

**FIG 2:**
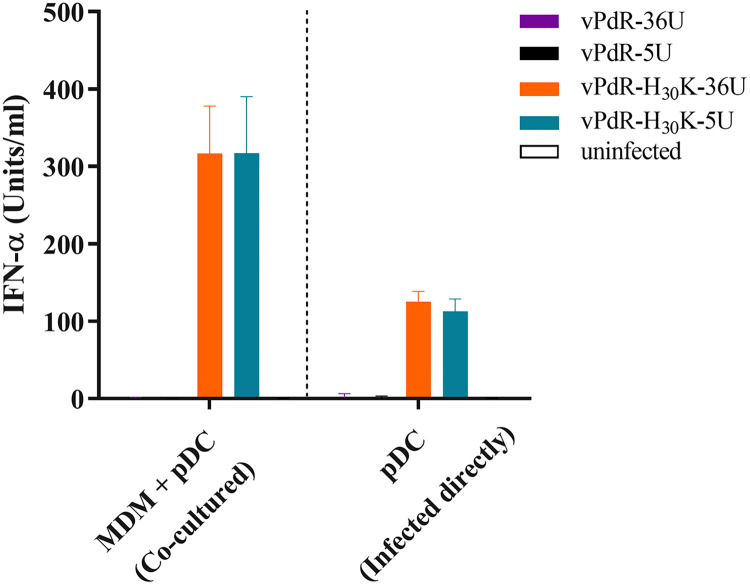
IFN-α induction in pDC by direct infection with virus or by coculture with preinfected MDM. Shown are mean IFN-α concentrations in cell culture supernatants from enriched pDC activated with MDM preinfected with vPdR-36U, vPdR-5U, vPdR-H_30_K-36U, or vPdR-H_30_K-5U or left uninfected (cocultured) or from enriched pDC infected directly with the indicated viruses. IFN-α was analyzed by ELISA and quantified based on porcine IFN-α of known bioactivity. The values represent the means of 3 replicate experiments, with the error bars showing the standard deviations.

It was shown previously that E^rns^ RNase activity could prevent IFN-α production by pDC upon contact with CSFV-infected cells (17, 18). In this study, the PdR strain that lacked E^rns^ RNase activity resulted in strong activation of pDC, and significantly stronger IFN-α production was observed when the pDC were stimulated by contact with infected MDM compared with direct infection. Remarkably, the length of the poly-U sequence of the 3′ UTR had no effect on the level of IFN-α induction in pDC (Fig. 2). This was observed both in the MDM-pDC coculture setup and after direct infection of pDC. The two viruses with functional E^rns^, vPdR-36U and vPdR-5U, did not induce any detectable IFN-α levels in pDC, either by direct infection or by contact with infected MDM (Fig. 2).

### The double mutant vPdR-H_30_K-5U induced acute CSF with high mortality in 5-day-old piglets.

**FIG 3:**
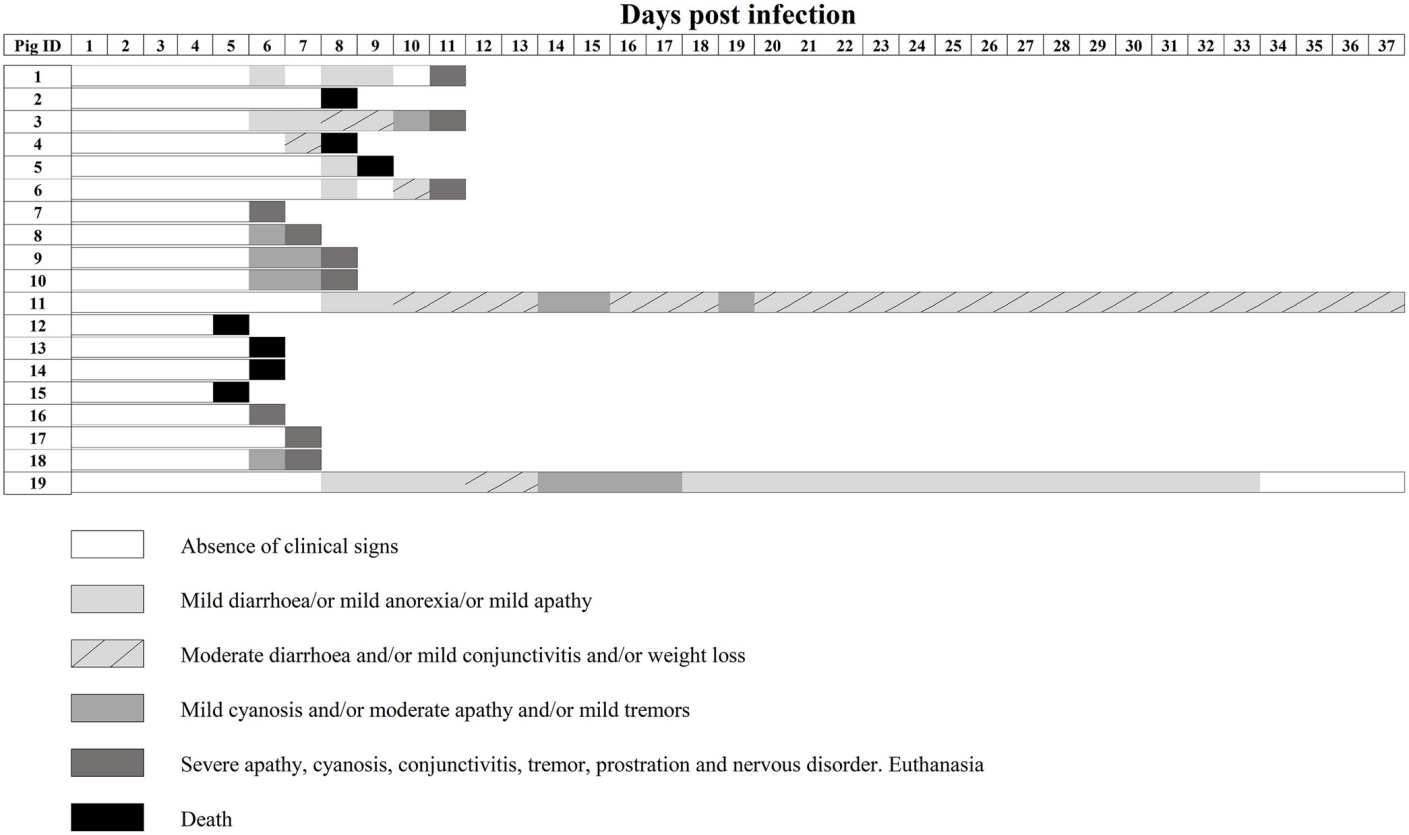
Individual clinical signs observed in piglets infected at the age of 5 days with vPdR-H_30_K-5U. Pigs 1 to 19 were inoculated intranasally with vPdR-H_30_K-5U, and the clinical signs were monitored daily during the whole study. The severity of the clinical signs is depicted according to the key.

Previous studies have shown that the sole removal of the poly-U insertion (vPdR-5U), unique in the PdR strain, enhanced CSF virulence in piglets compared to the case with the parent vPdR-36U (16). Meanwhile, virulence of the PdR strain was attenuated by abrogating the E^rns^ RNase activity alone (vPdR-H_30_K-36U) (17). In this study, we analyzed the phenotype of the double mutant vPdR-H_30_K-5U in 5-day-old piglets following the same experimental design as previously described (15, 16). Unexpectedly, 7 out of 19 infected piglets were found dead in the pen between days 5 and 9 after vPdR-H_30_K-5U infection. Ten additional piglets were euthanized between days 6 and 11 postinfection due to the severity of clinical signs, including prostration, weakness of hindquarters, moderate to severe conjunctivitis, cyanosis, tremor, and severe nervous disorders (Fig. 3). The two last piglets of the group (numbers 11 and 19) survived with moderate to mild clinical signs, including light diarrhea, weight loss, and in some cases conjunctivitis, from 8 days postinfection (dpi) until the end of the study (Fig. 3). Thus, the overall lethality of vPdR-H_30_K-5U in newborn piglets was 89.5%.

### Infection of 5-day-old piglets with vPdR-H_30_K-5U resulted in high virus load in tonsils despite low viremia and limited virus secretion.

**FIG 4:**
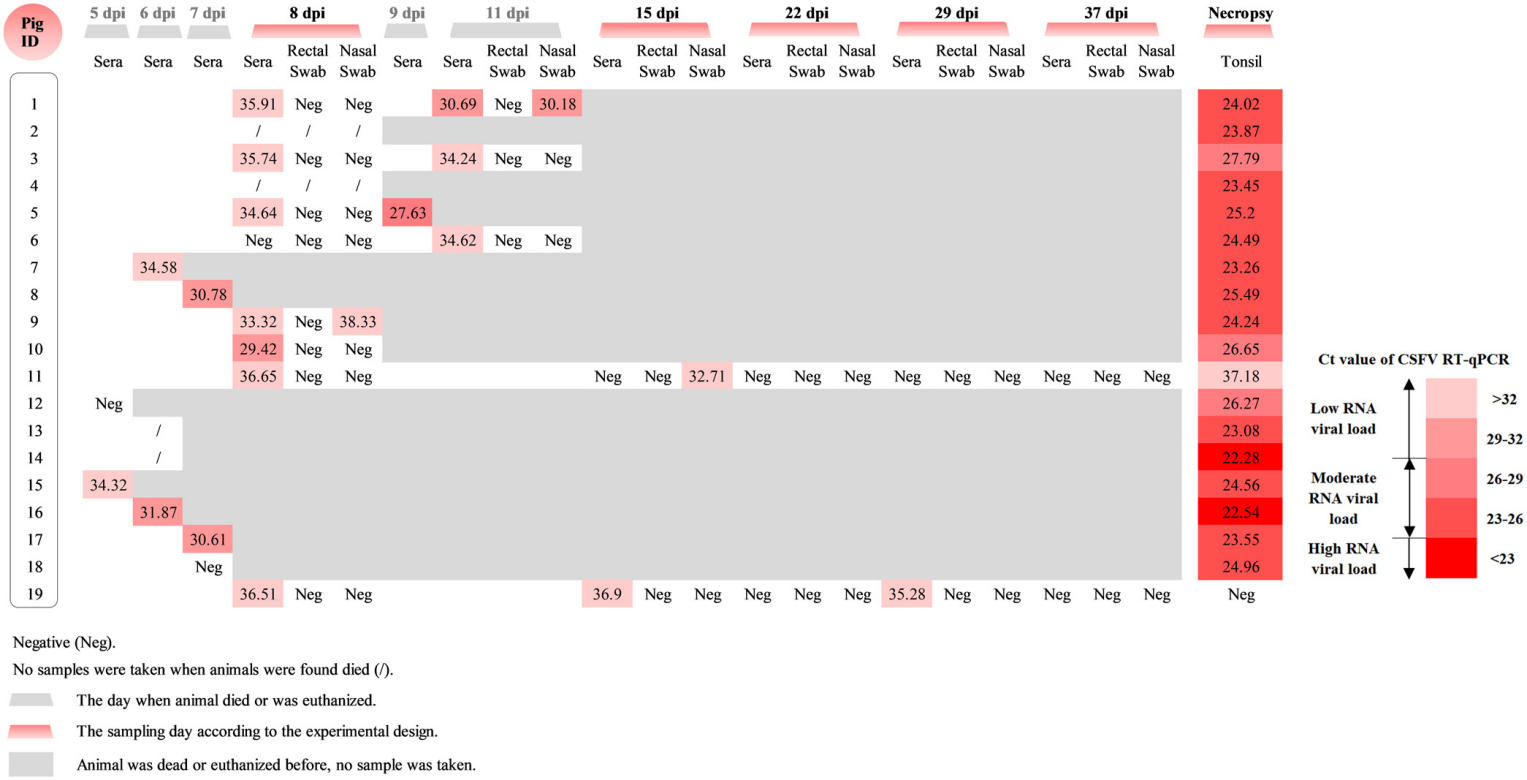
CSFV RNA detection in sera, swabs, and tonsils from the piglets infected with vPdR-H_30_K-5U. The CSFV RNA levels were determined by RT-qPCR in the sera and swabs at the indicated sampling time points and in the tonsils obtained at necropsy. *C_T_* values above 40 were considered negative.

We have previously reported that the loss of the poly-U insertion in the parental vPdR-36U enhanced CSFV replication in piglets (16). In contrast, vPdR-H_30_K-36U lost its ability to replicate in piglets (17). In present study, we evaluated the vPdR-H_30_K-5U double mutant for replication capacity *in vivo*. Serum samples could be collected from 7 out of the 11 vPdR-H_30_K-5U infected piglets that died or had to be euthanized before 8 dpi as described above. Five of them were CSFV positive, showing cycle threshold (*C_T_*) values between 30.61 and 34.58. At 8 dpi, seven out of the eight surviving pigs had a low serum CSFV loads, with *C_T_* values ranging from 29.42 to 35.91 (Fig. 4). After this time point, four animals either died or were euthanized from 9 to 11 dpi, showing *C_T_* values between 27.63 and 34.62 in the serum samples. Interestingly, one of these eight piglets (number 6) had a weak quantitative reverse transcription-PCR (RT-qPCR) signal (*C_T_* of 34.62) in the serum on day 11 only, despite severe clinical signs requiring euthanasia. Only two animals survived until the end of the trial (piglets 11 and 19), with low to undetectable CSFV RNA in the serum. Pig 11 had a weak RT-qPCR signal in the serum on day 8 only, while pig 19 was weakly positive on days 8, 15, and 29 (Fig. 4). Accordingly, the piglets did not secrete the virus in the feces or through the nose, except for two pigs with severe clinical signs (numbers 1 and 9) that were positive for CSFV by nasal swabs on days 11 and 8, respectively (Fig. 4). In addition, one of the two survivor pigs (number 11) was positive by nasal swab on day 15.

Interestingly and in contrast to the low CSFV load in the serum, all the tonsil samples collected from the 17 piglets that were euthanized due to severe CSF or were found dead were RT-qPCR positive for CSFV RNA, with moderate to high viral loads (Fig. 4). The two surviving piglets euthanized at 37 dpi were weakly positive in the tonsil, showing a *C_T_* value of 37.18 at that time (piglet 11), while piglet 19 was negative (Fig. 4).

Importantly, sequence analyses of the virus recovered from tonsils confirmed the expected 3′ UTR sequence and RNase knockout sequence in E^rns^, without any accidental mutation in these two genome regions (data not shown).

### Piglets that survived vPdR-H_30_K-5U infection seroconverted after 3 weeks.

**FIG 5:**
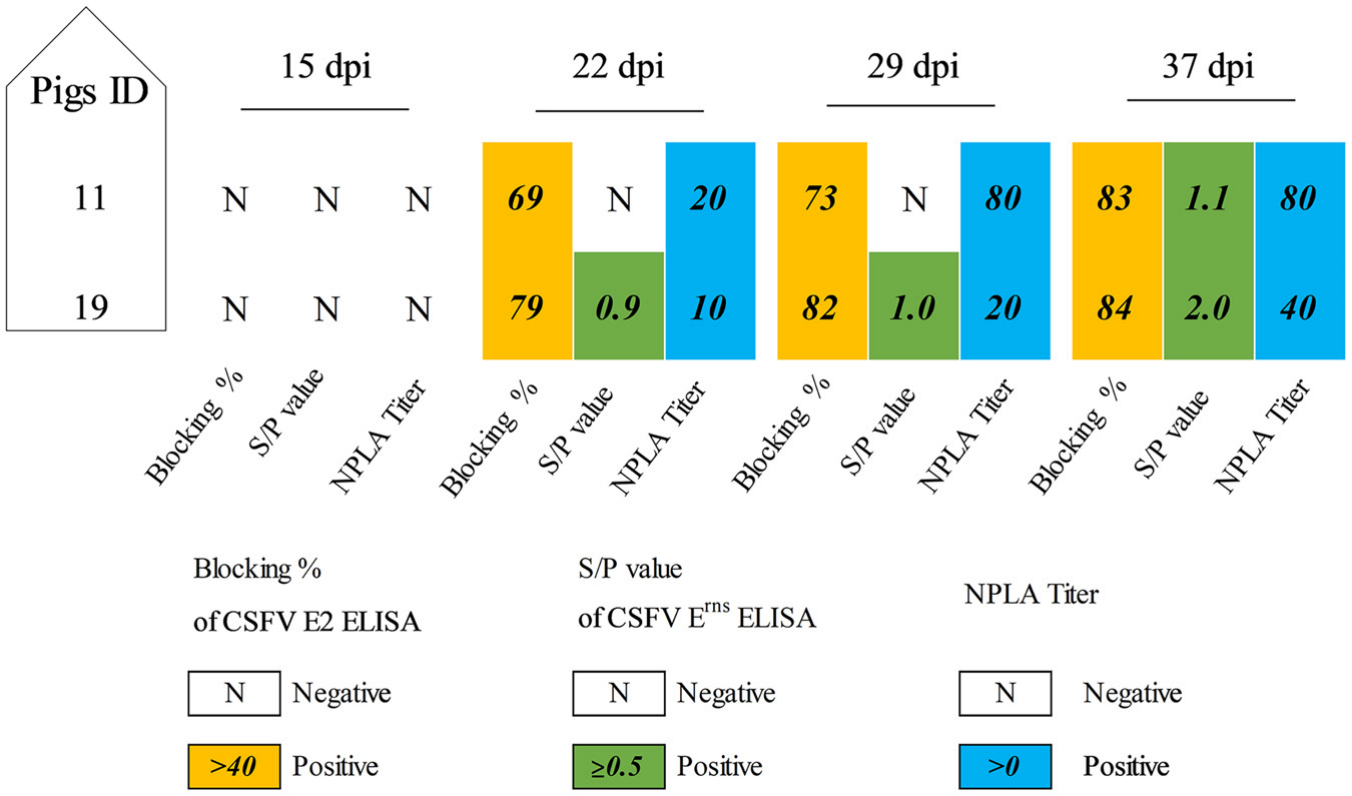
Evaluation of the humoral immune response in piglets after vPdR-H_30_K-5U infection. The E2- and E^rns^-specific antibody responses were measured by specific ELISAs and are shown as percent blocking or as S/P ratios. The neutralizing antibody titers were determined by the NPLA at weekly intervals. The cutoff values for positive samples are indicated for the respective test.

In accordance with previous work, infection with vPdR-H_30_K-36U resulted in enhanced neutralizing antibody responses compared with the case with the parental vPdR-36U in pigs (16, 17), while pigs infected with vPdR-5U showed a weak capacity to develop antibodies (16). In this study, the humoral response generated *in vivo* after infection with vPdR-H_30_K-5U was determined. The 2 out of 19 piglets that survived vPdR-H_30_K-5U virus infection developed CSFV E2-specific antibodies after 15 dpi, with the first positive values measured on day 22 (Fig. 5). One of them was also positive for E^rns^ antibodies from 22 dpi onwards, while the other was anti-E^rns^ positive for the first time on day 37, i.e., at the end of the study (Fig. 5). Low CSFV-neutralizing antibody titers were detected in the two animals on day 22, reaching values of 1:20. Likewise, a slight increase in the neutralizing antibody titers was noted for both animals at the end of the study, with values of 1:80 and 1:40 (Fig. 5).

### The vPdR-H_30_K-5U double mutant induced stronger IFN-α, IL-8, and IL-12 responses than its parent viruses.

To assess the role of the RNase activity and the 3′ UTR poly-U insertion in immune activation, the cytokine profiles of 14 serum samples from 5-day-old piglets infected with the vPdR-H_30_K-5U double mutant were evaluated using the Luminex system. Twelve samples were from animals that died or developed severe disease, collected from 5 to 8 dpi, and the other two samples were from the surviving pigs at 8 dpi. These sera were compared with samples from age-matched piglets infected with cDNA-derived wild-type or mutant PdR virus from previous studies.

**FIG 6:**
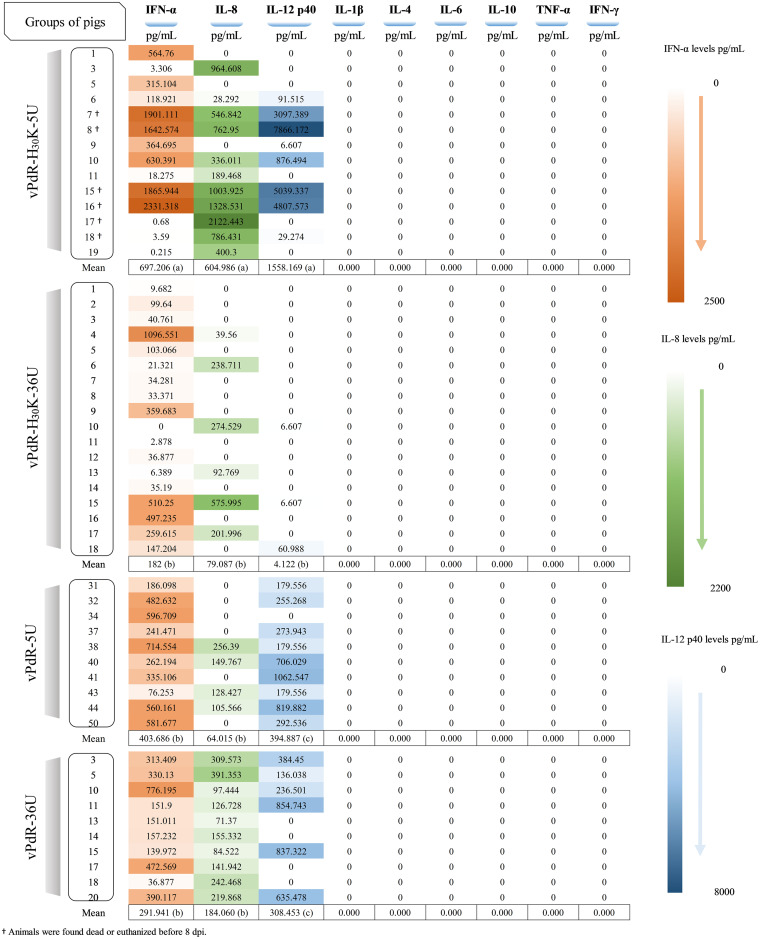
Cytokine levels in serum samples from vPdR-36U-, vPdR-5U-, vPdR-H_30_K-36U-, and vPdR-H_30_K-5U-infected piglets between days 5 and 8 postinfection. Cytokine concentrations are expressed as picograms per milliliter, calculated according to a standard curve. IFN-α, IL-8, and IL-12 levels from low to high are represented on a scale from light to deep orange, green, and blue, respectively. The mean value was determined for each cytokine per group. For each cytokine, the results from statistical comparison of the means between the groups is shown with letters in brackets, with different letters meaning significant differences between the groups (*P* ≤ 0.003), while the same letters show that there is no significant difference.

Remarkably, the highest IFN-α levels were detected in the serum samples of animals infected with vPdR-H_30_K-5U that were found dead or had to be euthanized from 5 to 11 dpi (Fig. 6), with IFN-α concentrations ranging from 315 to 2,331 pg/mL. These values were significantly higher than in the other three groups (*P* ≤ 0.001). For IL-8, the serum concentrations were also significantly higher in the piglets infected with vPdR-H_30_K-5U than in the other groups (*P* ≤ 0.003), with values between 28.292 and 2,122.44 pg/mL. Notably, the four piglets with the highest serum IFN-α levels after infection with vPdR-H_30_K-5U (numbers 7, 8, 15, and 16) also had IL-12 levels exceeding 3,000 pg/mL. This contrasted with the absence of any IL-12 response in the vPdR-H_30_K-36U group (three background signals; *P* ≤ 0.001) (Fig. 6). Interestingly, the two piglets that survived vPdR-H_30_K-5U infection showed background levels of IFN-α and IL-12 and low IL-8 levels. Of note is the complete absence of any detectable tumor necrosis factor alpha (TNF-α), IL-1β, IL-4, IL-6, IL-10, or IFN-γ signal in any of the serum samples analyzed, which was validated with the positive controls.

### In 3-week-old pigs, the double mutant vPdR-H_30_K-5U induced a milder disease with lower lethality than in 5-day-old piglets.

**FIG 7:**
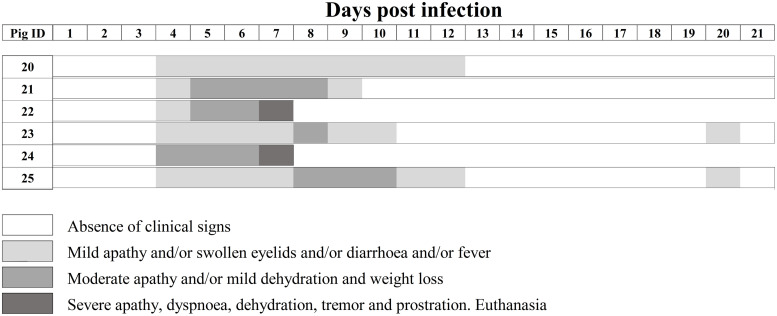
Individual clinical signs observed in pigs infected at the age of 3 weeks with vPdR-H_30_K-5U. Pigs 20 to 25 were inoculated intranasally with vPdR-H_30_K-5U, and the clinical signs were monitored daily through the whole trial. Different shades of gray represent the severity of the clinical signs according to the key.

We repeated the experiments by infecting 3-week-old weaned pigs with vPdR-H_30_K-5U, to clarify whether the phenotypes described above related to RNase-inactive E^rns^ in the absence of the poly-U sequence in the 3′ UTR of PdR are restricted to newborn piglets. The pigs were monitored for 21 days after infection and developed disease starting with apathy from day 4 after infection onwards. The clinical picture worsened for two of the animals, which had to be euthanized on day 7 (pigs number 22 and 24) after they had constantly elevated body temperature and developed severe apathy, dehydration, and prostration (Fig. 7). The remaining pigs showed reduced liveliness and suffered from conjunctivitis, diarrhea, and weight loss but recovered from 10 to 12 dpi until the end of the trial (Fig. 7). This corresponds to a 33% lethality. This is significantly different from the 89.5% lethality of the same virus in newborn piglets.

### In 3-week-old pigs, the vPdR-H_30_K-5U mutant resulted in high virus loads in tonsils with limited viremia and secretion as in 5-day-old piglets.

**FIG 8:**
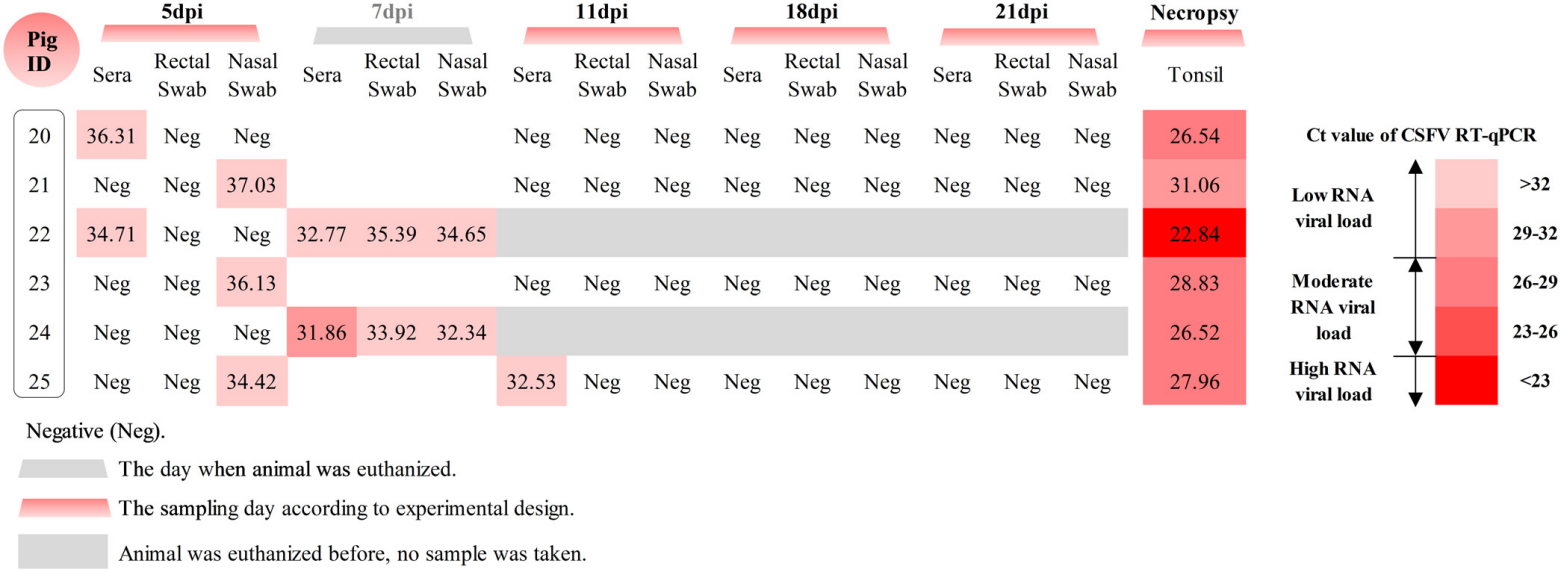
Determination of CSFV RNA in sera, swabs and tonsils from pigs infected at the age of 3 weeks with vPdR-H_30_K-5U. CSFV RNA was determined by RT-qPCR in the sera and swabs at different sampling time points and from the tonsil samples obtained at necropsy. *C_T_* values above 40 were considered negative.

As for the piglets, we analyzed the course of viremia, nasal and rectal virus secretion, and viral RNA in the tonsils of the 3-week-old pigs (Fig. 8). On day 5 after infection, two out of the six pigs were positive for CSFV RNA in serum, with *C_T_* values of 34.71 and 36.31, suggesting low viral loads (pig numbers 20 and 22). On the same day, three of the four pigs (numbers 21, 23, and 25) that had undetectable viremia were positive by nasal swabs only, while the last pig was still completely negative by all samples. At 7 dpi, the two pigs that had to be euthanized were also positive by sera and swabs. Subsequently, among the remaining 4 pigs that survived, only one animal tested weakly positive for CSFV by serum on day 11, while all remaining pigs were negative by serum and swab samples until the end of the study (Fig. 8).

The two pigs that had to be euthanized 7 dpi showed moderate to high viral RNA loads in the tonsils (Fig. 8). The four remaining animals showed low to moderate CSFV RNA loads in the tonsils at the end of the study 21 dpi. In addition, as in the previous experiment with the newborn piglets, sequence analyses of the virus recovered from the tonsils confirmed the integrity of the 3′ UTR sequence and the RNase knockout sequence in E^rns^ (data not shown).

### The 3-week-old pigs that survived vPdR-H_30_K-5U infection seroconverted from day 11 postinfection.

**FIG 9:**
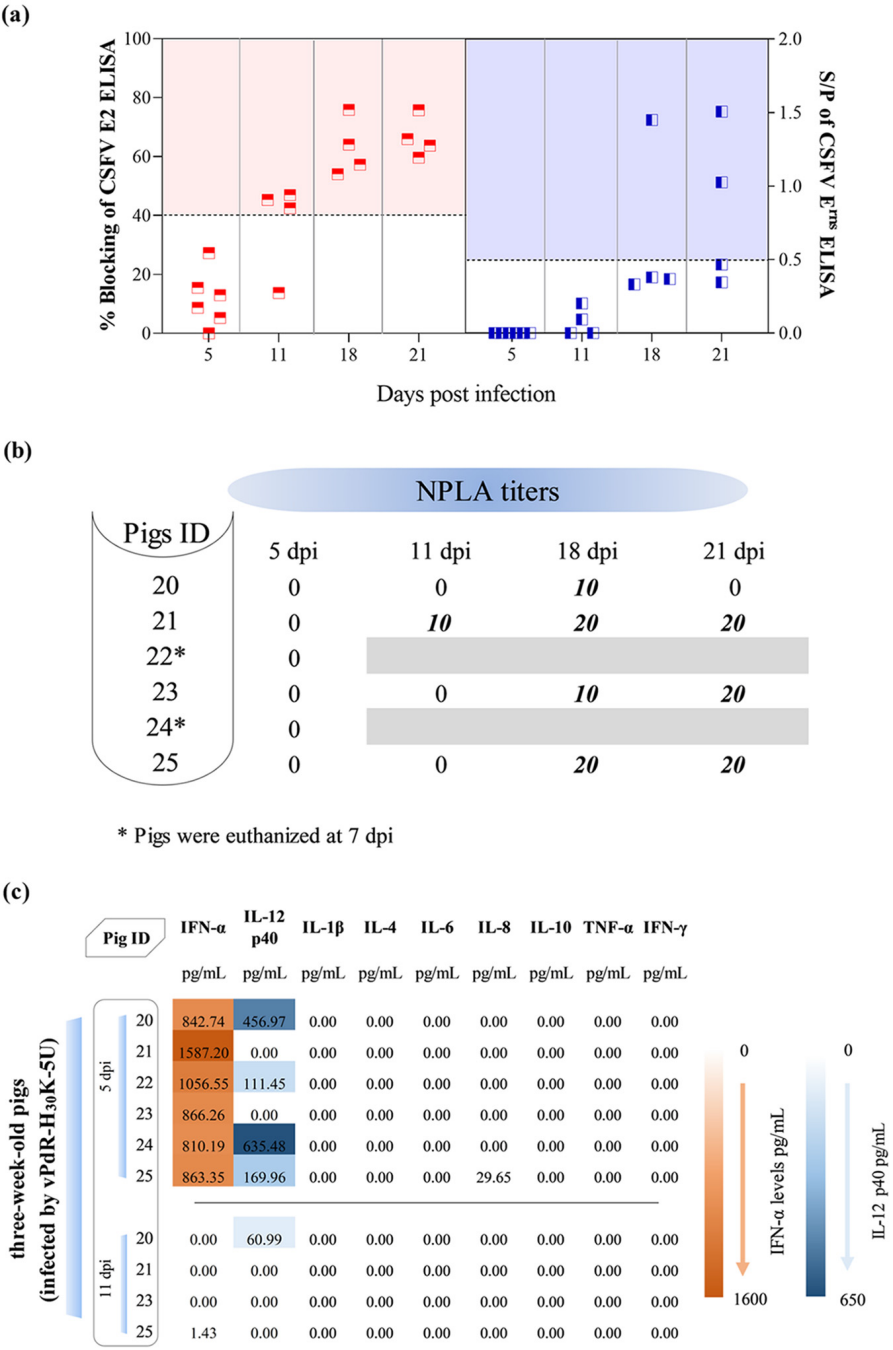
Evaluation of the humoral and innate immune responses in pigs infected at the age of 3 weeks with vPdR-H_30_K-5U. (a) Anti E2- and E^rns^-specific antibody responses are shown as percentage of blocking (left *y* axis, red squares) and S/P ratios (right *y* axis, blue squares), respectively, as measured by specific ELISAs. For E2 antibodies, blocking values between 30 and 40% were considered doubtful, and values equal or greater than 40% were considered positive (red shaded area). For E^rns^ antibodies, S/P ratios between 0.3 and 0.5 were considered doubtful, while S/P ratios equal or greater than 0.5 were considered positive (blue shaded area). (b) Neutralizing antibody titers determined by the NPLA. (c) Cytokine levels in serum samples at 5 and 11 dpi. IFN-α and IL-12 levels from low to high are represented on a scale from light to deep orange and blue, respectively.

CSFV-specific antibodies against the E2 glycoprotein were detected on day 11 after infection in three out of the four pigs that survived vPdR-H_30_K-5U infection (Fig. 9a). At 18 dpi, the four pigs had seroconverted. Meanwhile, the anti-E^rns^ antibody response could not be detected on day 11, and only one pig seroconverted against E^rns^ at 18 dpi, while a second pig became positive on day 21 (Fig. 9a). Notably, neutralizing antibody responses were detected in the surviving animals mainly after 18 dpi. However, the neutralizing titers never reached values over 1:20 (Fig. 9b). As expected, the two animals that were euthanized at 7 dpi (pigs 22 and 24) were negative for CSFV antibody responses (data not shown).

### In 3-week-old pigs, the vPdR-H_30_K-5U double mutant induced drastic elevation of IFN-α and IL-12, without IL-8 induction.

The cytokine profile on day 5 after infection of the 3-week-old pigs with the vPdR-H_30_K-5U double mutant was like the profile observed 5 to 8 dpi in 5-day-old piglets, except in the case of IL-8, which remained mostly undetectable in the older pigs. All six pigs showed high concentrations of serum IFN-α at 5 dpi, with values ranging from 810.19 to 1,587.20 pg/mL. Interestingly, a steep decrease in the IFN-α levels was registered at 11 dpi, and all the serum samples became negative in the surviving pigs (Fig. 9c). For IL-12, the concentrations were lower than in newborns, with values between 111.45 and 635.48 pg/mL detected in four out of six pigs at 5 dpi (Fig. 9c). Like IFN-α, IL-12 decreased at 11 dpi, being undetectable in three out of the four surviving animals (Fig. 9c). As for the newborn piglets, TNF-α, IL-1β, IL-4, IL-6, IL-10, and IFN-γ remained undetectable at the times analyzed (Fig. 9c).

## DISCUSSION

The present study shows, for the first time, a synergistic effect of lacking both the E^rns^ RNase activity and the 3′ UTR poly-U insertion in a low-virulence CSFV strain to increase virulence and CSF severity in pigs. Infection with the vPdR-H_30_K-5U double mutant induced acute disease with a high lethality in 5-day-old piglets as opposed to the parent low-virulence PdR strain characterized in previous studies. The low virulence of PdR was observed with wild-type parent virus in the field and demonstrated with clone-derived virus (vPdR-36U) (15, 16, 19). Of note, deletion of the long 3′ UTR poly-U sequence alone (vPdR-5U) partially enhanced virulence, while abrogation of the E^rns^ RNase activity alone (vPdR-H_30_K-36U) reduced virulence and *in vivo* replication (16, 17). Despite these differences, similar replication kinetics were observed in MDM infected with these four viruses (see also references 16 and 17), which altogether point to relevant virus-host interactions during CSFV replication *in vivo* that are not reflected in cell culture.

A high lethality, up to 89.5%, was observed in the vPdR-H_30_K-5U-infected 5-day-old piglets between 5 and 11 dpi. Likewise, the clinical signs recorded for the infected piglets were typical for the acute form of CSF, with rapid onset of disease and a lethal outcome within a few days (1). Meanwhile, the clinical signs developed by the two surviving piglets were associated with chronic CSF disease (1). In 3-week-old pigs, clinical signs of CSF were observed earlier (4 dpi) but were overall milder. Nevertheless, two of six pigs were euthanized for animal welfare reasons at 7 dpi. The rest of the infected animals (66%) recovered. These results support (i) age-dependent differences in CSFV susceptibility and disease severity (14, 20, 21) and (ii) a role for the E^rns^ RNase activity and the 3′ UTR in modulating the CSFV pathogenesis in pigs (16, 17).

Neutralizing antibodies play an essential role in protection against CSFV infection (1). The surviving animals of both ages developed specific anti-E2 and -E^rns^ antibodies after infection with vPdR-H_30_K-5U. However, the neutralizing antibody titers were lower than the threshold established for protection against CSFV infection at 3 weeks postinfection in both experimental groups (22). This may have led to the possible chronic outcome in the surviving animals (23). Thus, while the abrogation of the E^rns^ RNase activity in the vPdR-36U backbone enhanced the antibody responses (17), additional removal of the long poly-U sequence of the 3′ UTR resulted in the opposite effect, with reduced adaptive immune response in terms of neutralizing antibodies.

The results from present study showed a synergistic effect after knocking out the E^rns^ RNase and deleting the long 3′ UTR poly-U sequence in the PdR strain on CSF exacerbation and on increased CSFV replication in the tonsils, independently of the age of the pigs. The epithelial cells of the tonsillar crypts are described as primary sites of CSFV replication, preceding the viremic phase (1). For pestiviruses and flaviviruses, the 3′ UTR is a critical element influencing viral RNA replication (24–26). It is likely that the loss of the long poly-U in the 3′ UTR improves the capacity of the virus to replicate in the target cells of the tonsils (16). Contrasting with high replication in the tonsils, vPdR-H_30_K-5U infection resulted in mild viremia and low virus secretion. This may be a consequence of immune activation due to the lack of the E^rns^ RNase activity in the vPdR-H_30_K-5U mutant (17), favoring IFN-α responses at the systemic level, in the tissues where virus replicates before the viremic stage (27). This disproportional activation of innate immunity might limit systemic viral spread from the tonsils and increase virus clearance, and at the same time, it might induce strong cytokine-mediated symptoms. In addition, IFN-α overexpression may induce severe lymphopenia and lead to hematologic disruption (10, 27), which could also increase CSF severity.

The double mutant vPdR-H_30_K-5U resulted in significantly higher IFN-α induction in piglets than the single mutants or the parent vPdR-36U characterized previously (Fig. 6). High levels of systemic IFN-α were observed repeatedly in pigs infected with high-virulence CSFV (28, 29), which appears to be a hallmark of acute severe CSF (1, 10, 27). Contrasting with the differences in IFN-α induction by vPdR-H_30_K-5U and vPdR-H_30_K-36U *in vivo* (Fig. 6), similar levels of IFN-α were found in infected pDC (Fig. 2). This confirms the role of CSFV E^rns^ in the inhibition of IFN-α induction *in vivo* (17) and in pDC cultures (18), and it suggests a contribution of the 3′ UTR to innate immune activation *in vivo* but not in MDM-pDC cocultures. Of note, none of the mutations in PdR affected replication in MDM (Fig. 1a). Thus, the differences in IFN-α induction observed *in vivo* with vPdR-H_30_K-5U and vPdR-H_30_K-36U are more complex than simply a consequence to the basic replicative capacity of the virus.

Besides IFN-α, IL-12 was also drastically elevated in pigs that died or were euthanized after vPdR-H_30_K-5U infection, both newborn and older pigs. Interestingly, IL-8 was elevated in the infected newborn piglets only (Fig. 6 versus Fig. 9c). These results, together with the fact that IL-1β, IL-4, IL-6, IL-10, TNF-α, and IFN-γ could not be detected at the systemic level after vPdR-H_30_K-5U infection, suggest that IFN-α, IL-12, and to some extent IL-8 are the major cytokines contributing to CSF pathogenesis. A cytokine storm has been associated previously with lethal forms of CSF due to high-virulence CSFV, but this phenomenon has not been extensively studied (1). It should be noted that high levels of IL-8 and IL-12 were described for humans with a fatal outcome of influenza A virus (H5N1) or with severe infection with severe acute respiratory syndrome coronavirus 2 (SARS-CoV-2) (30–33). Likewise, high levels of IL-8 have been also detected in pigs suffering from severe African swine fever (34).

The present study shows for the first time the cooperative effect of the 3′ UTR and the E^rns^ RNase function in regulating the innate and adaptive host immunity against CSFV, favoring virus replication in target tissue and thus contributing to disease severity. In addition, these results highlight the role played by the long 3′ UTR poly-U insertion in CSFV attenuation and provide new insights for CSFV vaccine development.

## MATERIALS AND METHODS

### Cells and viruses.

The three cell lines used in this study, i.e., the porcine kidney cell line PK-15, the porcine aortic endothelial cell line PEDSV.15 (35), and the swine kidney cell line SK-6 (36), tested free of pestiviruses. The PK-15 cells, obtained from the ATCC (CCL-33), were grown in minimum essential medium (MEM) supplemented with 10% *Pestivirus*-free fetal bovine serum (FBS). The PEDSV.15 cells, provided by Jörg Seebach, University of Geneva, Switzerland, were grown in Dulbecco’s modified Eagle medium (DMEM) supplemented with sodium pyruvate, nonessential amino acids, 7% horse serum, and 2% porcine serum. The SK-6 cells were obtained from M. Pensaert, Faculty of Veterinary Medicine, Ghent, Belgium, and were cultured with the same medium as PEDSV.15 cells but without porcine serum. In addition, CD172a^+^ enriched porcine pDC and porcine MDM were prepared from specific-pathogen-free (SPF) pigs as described previously (16, 18, 37). For this, blood was collected from SPF pigs bred at the Institute of Virology and Immunology (IVI) in Mittelhäusern, Switzerland, in compliance with the animal welfare regulations of Switzerland under cantonal license BE127/2020.

CSFV strain Alfort/187, provided by the CSFV EU Reference Laboratory, Hannover, Germany, was used for virus neutralization assays. The cDNA-derived vPdR-36U, corresponding to the wild-type CSFV strain PdR, and the vPdR-5U virus lacking the poly-U sequence were from previous studies (16). The vPdR-H_30_K-36U virus, devoid of E^rns^ RNase activity, was also included in the present work (17). The cDNA-derived virus vPdR-H_30_K-5U was rescued from the respective cDNA as described below. All viruses were amplified by infecting cells with 0.1 50% tissue culture infectious dose (TCID_50_)/cell and harvested after 72 h. The virus titers were determined by endpoint dilution in SK-6 and PEDSV.15 cells and in porcine MDM using the peroxidase‐linked assay (PLA) (38). The virus titers, expressed in TCID_50_s per milliliter, were calculated using standard statistical methods (39).

### Construction of the infectious clone pPdR-H_30_K-5U.

The functional cDNA clone pPdR-H_30_K-5U, encoding RNase-inactive E^rns^ and 5 uridines in the 3′ UTR, was obtained by combining the plasmids pPdR-5U and pPdR-H_30_K-36U, described previously (16, 17). To this end, the SpeI-to-PspXI cassette of pPdR-H_30_K-36U was replaced with the corresponding cassette released from pPdR-5U, resulting in pPdR-H_30_K-5U. This construct was verified by Sanger sequencing.

### Virus rescue from cDNA.

The vPdR-H_30_K-5U virus was rescued from cDNA as described previously (16). Briefly, pPdR-H_30_K-5U was linearized with SrfI, and RNA was transcribed and purified. Then, 8 × 10^6^ PEDSV.15 cells were mixed with 1 μg of RNA in 0.4 mL of ice-cold phosphate-buffered saline (PBS) and electroporated with two pulses at 200 V and 500 μF in a 0.2-cm electroporation cuvette (Bio-Rad) using a Gene Pulser (Bio-Rad). The cells were then incubated in 75-cm^2^ flasks for 65 h at 37°C and 5% CO_2_. The rescued virus was passaged once in PEDSV.15 cells. In order to control the functionality of the constructs, the infectivity of the RNA transcripts was determined with an infectious-center assay as described previously (40). The complete genomes of the rescued viruses were determined by nucleotide sequencing to exclude any accidental mutation. Viral titers were determined in PEDSV.15 cells.

### Virus replication kinetics in cell culture.

Porcine MDM (500,000/well) were seeded in 24-well plates using serum-free medium. The cells were infected with vPdR-H_30_K-5U, vPdR-H_30_K-36U, and the parent vPdR-36U at a multiplicity of infection (MOI) of 0.005 TCID_50_/cell. After 1 h of incubation at 37°C, the inoculum was removed and the cells were washed once with serum-free medium followed by cultivation in complete culture medium at 37°C. At different times after infection, the plates were frozen at −70°C. After thawing, the supernatant was cleared from cell debris by low-speed centrifugation and the virus titers were determined by endpoint dilution in SK-6 cells and immunoperoxidase staining of CSFV E2 using the monoclonal antibody (MAb) HC/TC-26 (41).

### RNase activity assay.

Extracts of CSFV-infected SK-6 cells were analyzed for RNase activity as described previously (17, 18, 42). A 50-mer RNA probe (Dy-781-O1-RNA; prepared by Fabian Axthelm, Microsynth AG, Balgach, Switzerland) was mixed with the samples to be tested at a final concentration of 40 nM. RNase A (3 × 10^−3^ U/mL in MEM) was used as a digestion control, and 50 mM Tris HCl (pH 7.4) served as the negative control. After 45 min of incubation of the reactions at 37°C, 2 volumes of 97% formamide (Sigma) were added and a 10% polyacrylamide and 35% urea gel in 133 mM Tris HCl, 45.5 mM boric acid, and 3.2 mM EDTA was used for separation. Finally, the image was acquired with an Odyssey infrared imaging system (LI-COR).

### Western blot analyses.

Cells were lysed with a denaturing buffer (62.5 mM Tris HCl [pH 6.8], 2% sodium dodecyl sulfate [SDS], 10% glycerol, and 0.05% bromophenol blue) and the proteins were separated by 10% SDS-polyacrylamide gel electrophoresis under nonreducing conditions. The proteins were then transferred to polyvinylidene difluoride (PVDF) membranes (Immobilon-FL; Merck-Millipore) using standard protocols. The membranes were blocked with Odyssey blocking buffer (LI-COR) and incubated with the HC/TC-26 primary antibody and goat anti-mouse IRDye680 secondary antibody (LI-COR). An Odyssey infrared imaging system (LI-COR) was used for data acquisition.

### Stimulation of pDC for IFN-α production.

Enriched porcine pDC were stimulated with infected porcine MDM for IFN-α production as described previously (18). Briefly, porcine MDM were seeded in 24-well plates (200,000 cells/well) and infected at an MOI of 5 TCID_50_/cell for 24 h. The cells were washed four times, followed by addition of 10^6^ freshly isolated CD172a^+^ enriched pDC per well. The supernatants were harvested after another 22 h and analyzed for IFN-α production by enzyme-linked immunosorbent assay (ELISA). The infection of the MDM was verified by immunodetection of E2 using MAb HC/TC-26 and quantification of the immunoperoxidase signal at 450 nm.

### Experimental infection of 5-day- and 3-week-old pigs.

The experimental infection of pigs was designed following the protocol described previously, using animals from the same origin (16, 17). Nineteen newborn (5-day-old) piglets, born from *Pestivirus*-free sows, were allocated in the biosafety level 3 (BSL3) animal facilities at Instituto de Investigación y Tecnología Agroalimentaria, Centre de Recerca en Sanitat Animal (IRTA-CReSA; Barcelona, Spain). The piglets, numbered from 1 to 19, were inoculated intranasally with 2.5 × 10^4^ TCID_50_s of vPdR-H_30_K-5U (based on titers in PEDSV.15 cells). Nasal and rectal swabs and serum samples were collected from all animals at 8, 15, 22, 29, and 37 dpi. Tonsil samples were collected after euthanasia.

In addition, the virulence of the double mutant virus was also evaluated in six 3-week-old pigs from *Pestivirus*-free sows (numbered 20 to 25), as in previous studies (43, 44). The six pigs were inoculated intranasally with 2.5 × 10^4^ TCID_50_s of vPdR-H_30_K-5U. Nasal and rectal swabs and serum samples were collected at 5, 11, 18, and 21 dpi. Finally, tonsil samples were obtained after euthanasia.

The pigs were monitored daily in a blinded manner by a trained veterinarian as described before (45). When the animals reached the discontinuation criteria, euthanasia was performed with 60 to 100 mg of pentobarbital per kg of body weight injected in the vena cava cranialis, according to European directive 2010/63/EU. The endpoint criteria were moderate to severe clinical signs, including inability to drink or feed, fall of the hindquarters, prostration, and moderate to severe nervous disorders. The experiment was approved by the Ethics Committee from the Generalitat of Catalonia under animal experimentation project number 10514, in accordance with Spanish and European regulations, 21 June 2019.

### IFN-α quantification by ELISA.

IFN-α from pDC supernatants was quantified by ELISA as describe elsewhere (46, 47). The anti-pig IFN-α K9 and the F17 MAbs were provided by B. Charley, INRA, Jouy-en-Josas, France. Serial dilutions of recombinant IFN-α protein (PBL Biomedical Laboratories, Piscataway, NJ) served as a standard. The IFN-α concentrations (units per milliliter) in samples were determined by a regression curve based on optical densities of an IFN-α standard with known bioactivity.

### Detection of CSFV in infected pigs.

The CSFV RNA were extracted from sera, nasal and rectal swabs, and tissues from vPdR-H_30_K-5U-infected pigs by the MagAttract 96 *cador* pathogen kit (Qiagen), according to the manufacturer’s instructions. Tissue samples were ground in sterile Eagle’s minimal essential medium (EMEM; 1 g of tissue plus 9 mL of EMEM) supplemented with 2% penicillin (10,000 U/mL) and streptomycin (10,000 U/mL). RT-qPCR (48) was used to quantify the CSFV RNA in the collected samples. *C_T_* values equal or lower than 40 were considered positive. Samples that did not show any detectable fluorescence were considered negative. As previously quantified, *C_T_* values from 10 to 23 were defined as high, from 23 to 29 as moderate, and between 29 and 40 as low RNA viral loads (16, 49).

### Detection of antibody responses by ELISA and virus neutralization test.

The specific antibodies against CSFV E2 and E^rns^ in serum samples were analyzed using the CSFV Ab test (IDEXX Laboratories, Liebfeld, Switzerland) and pigtype CSFV E^rns^ Ab test (INDICAL BIOSCIENCE GmbH, Leipzig, Germany), respectively. For CSFV E2-specific antibodies, the results were represented as blocking percentage value, and samples with values of ≥40% were considered positive. Likewise, CSFV E^rns^-specific sample/positive (S/P) values of ≥0.5 were considered positive. Neutralizing antibodies were quantified with the neutralization peroxidase-linked assay (NPLA) against the Alfort/187 strain. The titers were expressed as the reciprocal dilution of serum that neutralized 100 TCID_50_s in 50% of the culture replicates.

### Detection of cytokines by Luminex assay.

The cytokines IFN-α, IL-1β, IL-4, IL-6, IL-8, IL-10, IL-12p40, IFN-γ, and TNF-α in serum samples were quantified with cytokine and chemokine 9-plex porcine ProcartaPlex panel 1 (Invitrogen, Bender MedSystems GmbH, Vienna, Austria) according to the manufacturer’s instructions. Results were acquired on a Luminex 200, and cytokine concentrations were expressed as picograms per milliliter, calculated according to a standard curve. A panel of serum samples from previous studies were included as control in the Luminex assay (16, 17). The panel included 10 serum samples from pigs infected with vPdR-36U and vPdR-5U (16), in addition to 18 serum samples from piglets infected with vPdR-H_30_K-36U (17), all collected at the end of the first week of infection.

### Statistical analyses.

Statistical analyses were performed using SPSS software, version 26.0 (IBM Corp., Armonk, NY), using “pig” as the experimental unit. Independent-sample *t* test was chosen to compare cytokine levels among all experimental groups. The significance level (α) was set at a *P* value of <0.05.
